# Green Tea Catechins as Therapeutic Antioxidants for Glaucoma Treatment

**DOI:** 10.3390/antiox12071320

**Published:** 2023-06-21

**Authors:** Tsz Kin Ng, Kai On Chu, Chi Chiu Wang, Chi Pui Pang

**Affiliations:** 1 Joint Shantou International Eye Center of Shantou University and The Chinese University of Hong Kong, Shantou 515041, China; micntk@hotmail.com; 2 Department of Ophthalmology and Visual Sciences, The Chinese University of Hong Kong, Hong Kong; 3 Department of Obstetrics and Gynaecology, The Chinese University of Hong Kong, Hong Kong

**Keywords:** green tea, EGCG, glaucoma, retinal ganglion cells, anti-oxidation, anti-inflammation

## Abstract

Glaucoma is the leading cause of irreversible blindness and visual impairment, affecting more than 80 million individuals worldwide. Oxidative stress and inflammation-induced neurodegenerative insults to retinal ganglion cells are the main pathogenesis of glaucoma. Retinal ganglion cells, the retinal neurons transmitting the visual signals to the visual cortex in the brain, have very limited regeneration or recovery capacity after damages. Apart from intraocular pressure-lowering treatments, there is still no clinically effective treatment to rescue the degeneration of retinal ganglion cells in glaucoma. Dietary antioxidants are easily accessible and can be applied as supplements assisting in the clinical treatments. Catechins, a chemical family of flavonoids, are the phenolic compounds found in many plants, especially in green tea. The anti-oxidative and anti-inflammatory properties of green tea catechins in vitro and in vivo have been well proven. They could be a potential treatment ameliorating retinal ganglion cell degeneration in glaucoma. In this review, the chemistry, pharmacokinetics, and therapeutic properties of green tea catechins were summarized. Research updates on the biological effects of green tea catechins in cellular and animal experimental glaucoma models were reviewed. In addition, clinical potentials of green tea catechins for glaucoma treatment were also highlighted.

## 1. Green Tea Catechins: Chemistry and Pharmacokinetics

### 1.1. Chemistry of Green Tea Constituents

**Figure 1:**
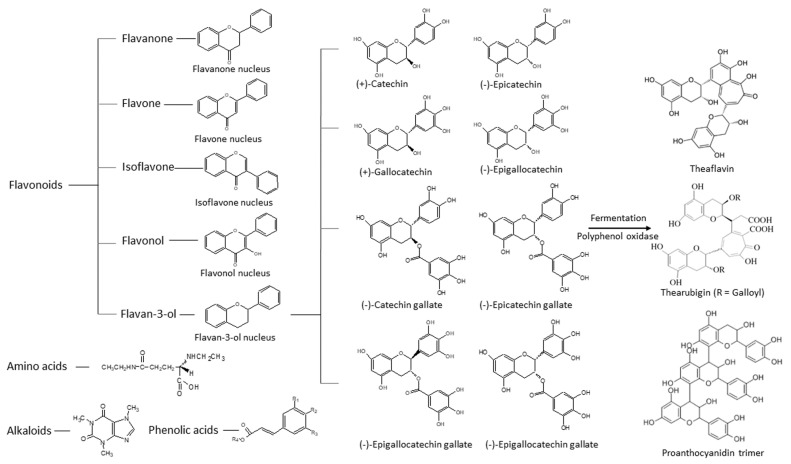
Structures of polyphenols, amino acids, and alkaloids that are present in green tea.

Tea is the most commonly consumed beverage worldwide. It comes from the leaves of the tea plant, *Camellia sinensis*. Different harvesting, manufacturing, and fermentation processes result in different types of tea, such as white, black, green, or oolong tea. Green tea is obtained by steaming and roasting fresh tea leaves under strictly controlled conditions so as to preserve the polyphenols from oxidation by polyphenol oxidase. Many constituents are present in the green tea infusion, including polyphenol polymers, amino acids, polysaccharides, saponins, alkaloids, and polyphenols (Figure 1). The compositions depend on the *Camellia* species, harvesting process, storage conditions, and processing methods. Polyphenol polymers, including theaflavins, thearubigins, and proanthocyanidin polymers, are oxidized and polymerized products of catechins monomers. Their anti-inflammatory and hepato-protective properties have been reported in experimental rodent models [1,2]. The concentrations of polyphenol polymers, theaflavins, and thearubigins are about 3–6% in green tea, 12–18% in black tea, and 8 to 20% of catechins in oolong tea, respectively. Amongst the tea amino acids, L-theanine is known to possess relaxation and cognitive improvement properties for humans [3]. Polysaccharides of glucose, galactose, rhamnose, and arabinose in tea are conjugated with different chemical groups resulting in diversified biological activities, including anti-oxidative and anti-diabetic activities [4,5]. Tea saponins, the natural non-ionic surfactants extracted from the aqueous layer, have displayed anticancer, antimicrobial, and cardiovascular protective properties in animal studies [6]. Benefits to human have also been shown [7]. Tea alkaloids, including caffeine, theobromine, and theophylline could improve cognition with antioxidative, anti-diabetic, and anti-obesity effects according to animal studies [8,9]. Polyphenols in tea have been the most extensively studied among the tea constituents due to their strong biological activity and high abundance [10]. Polyphenols in green tea are mainly flavonoids. Based on their nuclear structures, green tea flavonoids can be classified into flavanones, isoflavanones, flavones, flavonols, flavan-3-ols (catechins), and hydroxycinnamic acid. Amongst the polyphenols, catechins (flavan-3-ols) possess the most beneficial biomedical properties for human health [11]. The main catechins in green tea are as follows: (+)-catechin (C), (−)-epicatechin (EC), (−)-gallocatechin (GC), (−)-epigallocatechin (EGC), and their gallate derivatives, (−)-catechin-3-gallate (CG), (−)-epicatechin-3-gallate (ECG), gallocatechin-3-gallate (GCG), and (−)-epigallocatechin-3-gallate (EGCG), respectively (Figure 1). The flavan-3-ol concentration is about 50% in green tea and 10% in black tea, respectively.

**Figure 2:**
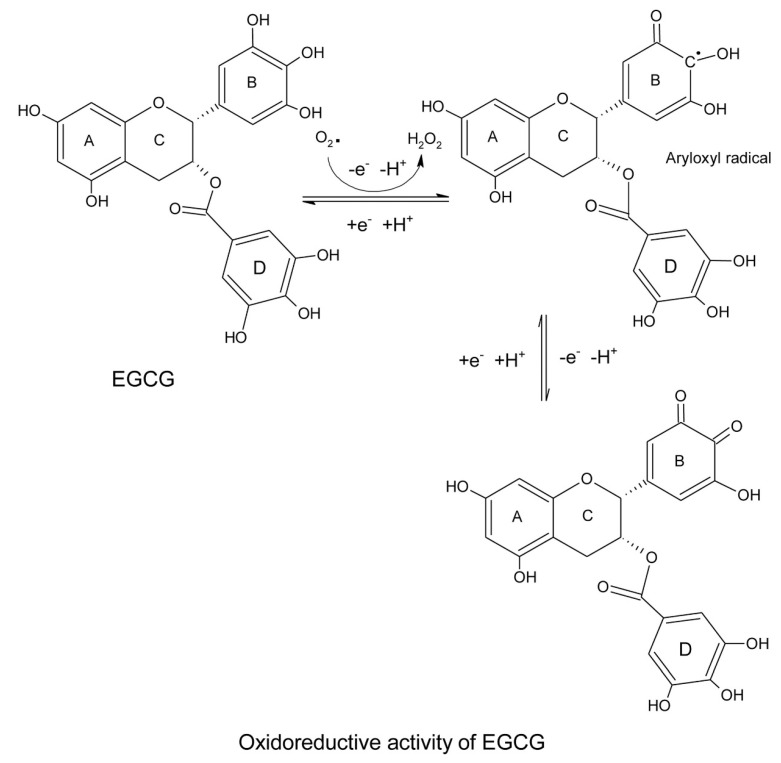
Resonance structures of epigallocatechin-3-gallate after reaction with reactive oxygen species.

EGCG is the most abundant and biologically active catechin with proven health-promoting properties [12,13]. Its biochemical activities are attributed to its structural moiety and hydroxyl groups [14]. EGCG has eight hydroxyl groups that contribute hydrogen radicals to reactive oxygen species and form stable resonance structures (Figure 2). Unlike the other flavonoids of green tea polyphenols, the pro-oxidant activity of catechins is relatively low as they do not have any double bonds in C2–C3, nor any ketone groups in C4 to form further resonance structures in the C ring [15]. Therefore, catechins can cross-link with each other to form stable polymers, such as thearubin [16]. Consequently, catechins are lower in toxicity compared toother tea polyphenols.

The standard reduction potential (E°) is correlated to the cellular antioxidant activity (CAA). Green tea catechins all have a lower reduction potential than the endogenous anti-oxidant glutathione (GSH), thereby being indicative of a higher antioxidant activity. GSH (0.310 V) < C (0.281 V) = EGC (0.287 V) < EC (0.277 V) < EGCG (0.104 V) < ECG (0.098 V) [17].

**Figure 3:**
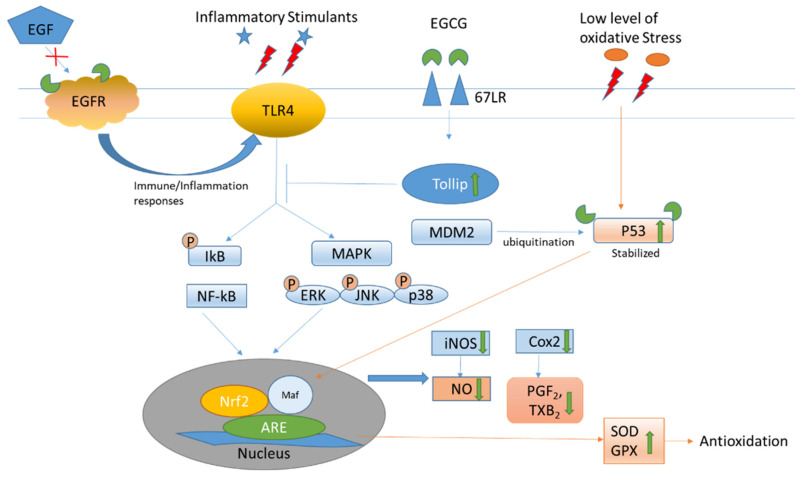
Epigallocatechin-3-gallate interacts with various receptor/mediators to relieve inflammation and oxidation. The green arrow indicates the effects of EGCG on the oxidative stress.

Besides its intrinsic antioxidative properties, the anti-inflammatory and antioxidative activities of EGCG have been attributed to its interaction with the cellular membrane protein receptor (Figure 3). It binds to 67 kDa laminin receptors (67 LR) to upregulate the Toll-interacting protein (Tollip). Increased Tollip expression induces negative effects of inflammatory associated Toll-like receptor (TLR) signaling, leading to the deactivation of the NF-kB and MAPK pathways, which act on anti-oxidant response elements (AREs) in the nucleus for both anti-inflammatory and anti-oxidant responses [18]. The subsequent lowering of inflammatory mediators, such as inducible nitric oxide synthetase (iNOS) decreases the production of reactive oxidative species. As the EGCG molecule possesses multiple hydroxyl groups, it is potent in binding through hydrogen bonding with amino acid residues including serine and tyrosine on the active sites of the membrane receptor to change the structural conformation and exert various signaling and biological activities. It interacts with the serine residue at the N-terminal domain of tumor suppressor p53, which is a sensor of oxidative stress, to change the structural conformation and inhibit the ubiquitination of p53 by murine double minute 2 (MDM2). The stabilized p53 can thus be retained for anti-tumor activities [19]. The pleiotropic effects of EGCG have also been attributed to its multiple binding properties. It can moderate the redox, inflammation, and cell cycle status through its multiple receptor affinities. It activates the epidermal growth factor (EGF) receptor in the absence of EGF but inhibits EGF-induced EGF receptor activation by affecting the topology of the EGF receptor transmembrane domain [20]. EGCG can also inhibit the activation of the wild-type and some mutants of the EGF receptor in non-small cell lung cancer cell lines [21].

Apart from binding to specific receptors, such as to 67LR for anti-inflammation, pro-oxidation of catechins generate reactive oxygen species (ROS) to act as secondary messengers to stimulate various signaling pathways, which may be mediated by receptors from the cell surface to the nucleus. For example, EGCG can bind to the active sites of thioredoxin (Trx) to inhibit the Trx/Trx receptor, which facilitates anti-oxidation to increase the ROS level [22]. ROS in turn can serve as an anti-bacterial agent [23]. EGCG activates calcium/calmodulin-dependent protein kinase β (CaMKKβ) to increase energy metabolism and elevate cytosolic calcium levels, thereby contributing to increase NO production [24]. It increases cyclic adenosine 5′ monophosphate (cAMP) in endothelial cells and platelets to promote the phosphorylation of eNOS and vasodilator=stimulated phosphoprotein [25] to cause vaso-relaxation [26]. Furthermore, it activates adenosine 5′ monophosphate-activated protein kinase (AMPK), which reduces endothelin-1 expression [27,28] to improve vasodilation [29] (Table 1).

### 1.2. Pharmacokinetics of Catechins in the Eye

Following oral administration, tea catechins are first absorbed by the small intestine, where they are conjugated with glucuronic acid, sulfate, or O-methylation before passing to the liver tissue cells for metabolism. Excess catechins are either secreted with the bile into the small intestine for the enterohepatic recirculation or pass into the colon for degradation by the resident microorganisms. The catabolites are either reabsorbed into plasma and excreted into the urine or passed out through the feces. The catechins, conjugates, and catabolites are distributed to various organs and tissues to exert various biological activities. As the bioavailability of catechins depends on its absorption and metabolism, the extensive metabolic processes render the levels of catechins to be very low, which is a limitation for antioxidative treatment.

The absorption efficiency of the catechins depends on the physicochemical properties, including molecular size, steric configuration, solubility, hydrophilicity, pKa, the presence of galloylated derivatives, and the presence of food matrix [30]. As the absorption involves efflux transporters, such as multidrug resistance-associated protein 2 (MRP2) in the small intestine [31], this results in a low bioavailability [32,33] and variability of the absorption rate. Co-administration of food and drugs can interact with the absorption of catechins [34]. The maximum plasma levels of free EGCG and EGC can increase more than 3.5-fold in the fasting condition [35]. When food is co-administrated with catechins, the time of maximum concentration (T_max_) of catechins would be prolonged for two times due to the gastric emptying rate slowing down. This rendered the maximum concentration (C_max_) of catechins to decrease by 3.5 times with breakfast. However, when catechins were co-administrated with carbohydrates, the oral bioavailability (AUC) of flavanol was found to have increased by 140% [36], which was deemed to be possible by suppressing the intestinal efflux and stabilizing the catechins in the lumen.

Catechin absorption is sterically and structurally dependent [37]. The levels of epi-isomers are higher than its enantiomers, EGC > GC, EC > C, and EGCG > GCG, respectively in the plasma of SD rats after oral administration. The plasma levels of non-gallated catechins, including EGC, GC, EC, and C, are higher than the gallated catechins, EGCG, GCG, and ECG. However, when green tea extract (GTE), with a higher proportion of EGCG is administered, the relative AUC of C was higher than that of EC, suggesting an unknown interaction between C and EGCG during absorption [38]. Although EGCG is a dominant component of green tea extract, its relative AUC level is low, indicating that the absorption ability of EGCG is poor.

**Figure 4:**
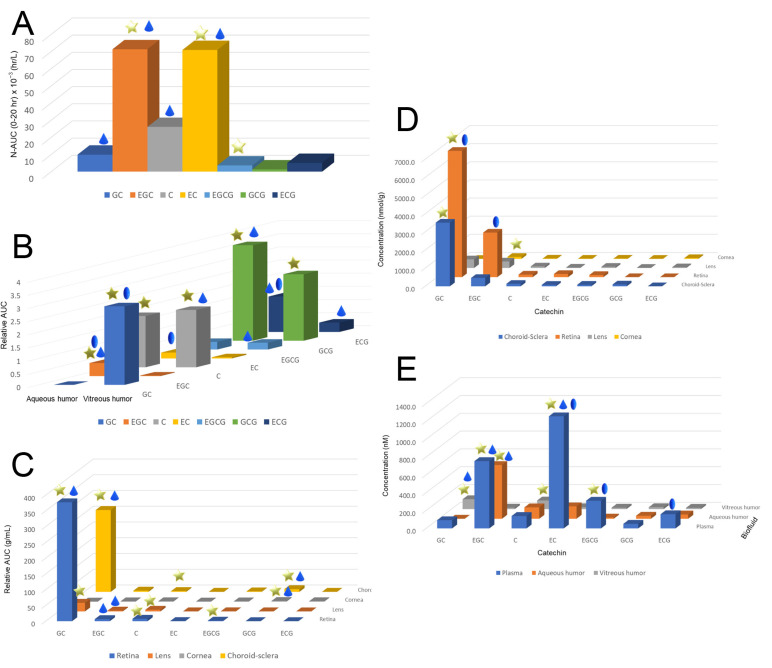
The exposure level, maximum concentration, and elimination of total catechins in the plasma, ocular fluid, and tissues of Sprague–Dawley rats. (**A**) Relative area under the curve (AUC) levels of different catechins in the plasma after normalization by the corresponding input catechin dose in the GTE. Non-gallated levels were higher than of the gallated derivatives while epimers were higher than the non-epimers. (**B**) Relative AUC levels of catechins in vitreous and aqueous humor. Vitreous humor was selective to non-epimer but showed no selectivity on gallated and non-gallated catechins. No particular trend of catechin selectivity appeared in the aqueous humor. (**C**) Relative AUC levels of catechins in the retina, lens, cornea, and choroid–sclera. (**D**) Maximum concentration of catechins in the plasma, aqueous and vitreous humors, and (**E**) eye tissues after a single dose of 550 mg/kg of Sunphenon DCF-1 green tea extract administrated orally to rats. Star: the level of an epimer was significantly higher than the corresponding non-epimer or vice versa in the same ocular compartment (*p* < 0.05); Droplet: the level of a catechin was higher than the corresponding gallate derivative or vice versa in the same compartment; Oval: the level of one of the catechins was significantly higher in one compartment than the other compartment (*p* < 0.05). GC: (−)-gallocatechin; EGC: (−)-epigallocatechin; C: (+)-catechin; EC: (−)-epicatechin; EGCG: (−)-epigallocatechin-3-gallate; GCG: gallocatechin-3-gallate; and ECG: (−)-epicatechin-3-gallate.

After a single dose administration of 550 mg/kg GTE into the SD rats, the ingested catechins are distributed across various ocular tissues, including aqueous humor, vitreous humor, choroid–sclera, retina, lens, and the cornea (Figure 4) [37]. The C_max_ of GC and ECG can reach a hundred micromolar levels in the choroid–sclera and retina, but only 1.5 μM in the lens, respectively (Table 2). These were the effective doses used in many in vitro studies. GTE can exert antioxidative, anti-inflammatory, and anti-apoptotic effects on the ocular tissues, especially for the retina [37,38,39]. Steric selectivity of distribution was also found in different ocular compartments. Vitreous humor was selective to non-epimer catechins but did not show a preference to the non-gallate derivatives preference as the plasma. Other ocular tissues did not show any steric and structural specificity except for the finding that GC was dominated. Catechins could also pass into various fetal tissues, including the eye [40]. However, the C_max_ levels of catechins were at the nanomolar level which may not be biologically effective. On the other hand, the C_max_ of EGCG in the fetal eye could reach to 0.83 μM which may therefore affect or benefit various tissue developments.

Catechins are mainly eliminated through urine and biliary excretion. More water-soluble non-gallated catechin derivatives, such as parent and conjugated compounds are mainly excreted in the urine, while major gallated catechins, which are less water-soluble, are excreted through the bile to the colon. A few epi- or gallocatechin-O-sulfate conjugates, but not the gallated catechin conjugates from ECG and EGCG, have been found in the urine [41]. This suggests that the gallated derivatives that undergo phase II metabolism are minimal. The levels of flavan-3-ol metabolites, mainly from (−)-epigallocatechin and (+)-gallocatechin, excreted into urine was calculated to be about 8.1–28.5% of the intake [42]. EGCG was excreted through the bile and eliminated through the feces but not through the kidneys [43], possibly due to the hydrophobic gallated catechins bound to plasma protein that limited renal excretion as a result [44,45].

The elimination rates of catechins in the ocular tissues were found to be higher than in the humors and plasma of SD rats [38]. The elimination rate of GC was from 0.2 h^−1^ to 2.4 h^−1^ in the retina, whereas the elimination rate of ECG in the vitreous humor was 0.04 to 0.2, respectively. On the other hand, the EGCG level can affect the elimination rates of other catechins in the ocular tissues (Table 2). Doubling the level of EGCG present can lower the elimination rates of other catechins, particularly in the retina, and aqueous and vitreous humors (Figure 4) [39]. Some active elimination or metabolic mechanisms, which can be affected by EGCG, could also arise in the ocular tissues. This mechanism could be associated with aqueous and vitreous humor elimination.

The elimination rates of catechins in the maternal plasma were faster than the fetal tissue. The elimination rates of GC and EC were 0.26 and 0.3 h^−1^ for the maternal plasma and 0.08 and 0.1 h^−1^ for the fetal kidney, respectively [41]. The fetal organs were not well developed for the elimination process. Similarly, the levels of GC and EGCG in the fetal eye were sustained at relative high levels (about 50 ρmol/g) without an apparent elimination during the studying period, while the elimination rate of EC was very slow (0.06 ± 0.06 h^−1^). It has been suggested that catechins can perfuse into the fetal eye and remain there for a long time.

Steric structures of catechins also affect the metabolism [46]. Equal quantities of (−)-EC, (−)-C, (+)-EC, and (+)-C fed to human males resulted in different bioavailabilities. Different levels of stereoisomers, including (−)-EC > (+)-EC > (+)-C > (−)-C, non-methylated conjugations, and 3′- and 4′-*O*-methylation of epimers were found in the plasma and urine. Also, the conjugation of gallate derivatives, including ECG and EGCG, were not found in the plasma and urine [47], which was deemed to probably be due to the inhibition of phase II enzymes by the gallated moiety of the catechins. The extensive metabolism and enzymatic resistance of some conjugates, including sulphates during sample processing can lead to large variations in pharmacokinetics [37].

## 2. Therapeutic Properties of Green Tea Catechins: Antioxidation and Anti-Inflammation in the Eye

Polyphenols, especially catechins, are known for their beneficial effects for health maintenance, along with their therapeutic effects [48]. These effects have been attributed to the powerful anti-oxidative and inhibition of lipid peroxidation through the chelation of metal ions to prevent oxidation reactions [49], and the hydroxyl groups for free radical scavenging. Therefore, the scavenging power of galloylated catechins, such as (−)-EGC, are stronger than non-galloylated catechins such as (+)-C [50]. The gallate derivative, (−)-EGCG, has the strongest radical scavenging capacity amongst the catechins [51]. Moreover, owing to the possession of a vicinal diol in the B-ring galloyl moiety, and an ortho-hydroxyl group in the A-ring, catechins can chelate the catalytic metal ions to generate free radicals. Since the hydroxyl groups in the catechins are essential for antioxidation, methylation can subsequently reduce the anti-oxidation power.

In addition to the radical scavenging process, catechins and their conjugates can cover or even incorporate themselves into the lipid membrane bilayer externally and internally to block the access of free radicals and stabilize the membrane through decreased lipid fluidity [52]. EGCG interacts with both the hydrophobic and hydrophilic regions of the lipid bilayers to protect the membrane from attack by the hydrophilic and hydrophobic oxidants [53]. Meanwhile, polyphenols can also induce various endogenous molecular pathways to activate the expression of antioxidant enzymes and suppress the pro-oxidative pathways. Catechins can activate glutathione S-transferase and deactivate xanthine oxidase and nitric oxide synthase, respectively [54]. More recently, the oral administration of EGCG to rats has been shown to increase ascorbic acid levels and oxygen radical absorbance capacity in the plasma [55].

Whilst the anti-oxidative effects of catechins have been attributed as beneficial health effects, the pro-oxidative effects and the subsequent stimulation of the relevant signaling pathways may account for the in vivo protection mechanisms. EGCG can be oxidized to produce hydrogen peroxide in cell culture medium, but these cellular actions can be abolished by SOD and catalase [56]. The anti-tumor activity caused by hydrogen peroxide generated from the pyrogallol moiety can reduce Fe (III) to Fe (II), triggering ROS production [57,58]. In an in vivo study, GTE, EGCG, EGC, and gallic acid showed pro-oxidative effects in that they significantly reduced GSH from 33.3–43.3% and increased GSSG, methemoglobin, and plasma hemoglobin in GPD-deficient erythrocytes, which are vulnerable to oxidative stress [59]. However, pro-oxidation has usually been demonstrated under experimental conditions and non-physiologically high concentrations under in vitro studies [58]. The concentration of EGCG and metabolites present in vivo (1–2 μM) can produce low levels of intracellular ROS to promote signal transduction pathways [27,60]. Moreover, GTE containing a high concentration of EGCG could increase oxidative stress in the plasma, aqueous humor, vitreous humor, cornea, and retina in SD rats even under lower physiological levels (<1 μM in plasma); yet, the 8-isoprostane level was lower than half of the EGCG level. GTE with a high EGCG content was found to induce superoxide dismutase 1 and glutathione peroxidase-3 expression, but also suppressed catalase in the retina. These pro-oxidation effects can occur at physiological level and is influenced by both chemical and biological activities of GTE, indicating that an optimal EGCG level is needed if GTE is used for health remedies [38].

The inhibition of inflammation was accompanied with the elevation of oxidative stress [61]. The increased ROS activates NF-κB and NF-E2-related factor 2 (Nrf2) to express the antioxidative factors HO-1 and glutathione [62]. In many antioxidative and anti-inflammatory studies, EGCG pre-treatment was required to protect against oxidative insult and inflammation induction [63]. We have proposed that the protective actions against oxidative stress and inflammation may be secondary to the induction of endogenous antioxidant proteins, as influenced by the pre-conditioned, pro-oxidative effects under physiological conditions [64].

Since EGCG can activate antioxidative nuclear translocation elements in the Nrf2/HO-1 pathway for both the antioxidative and anti-inflammatory responses, the antioxidation and anti-inflammation effects of catechins are always simultaneous as a result. In the retina of a rat model, GTE suppressed the activation of microglial cells, astrocytes, and Müller glia in a dose-dependent manner following lipopolysaccharide (LPS) induction. It also reduced the expression of the pro-inflammatory cytokines IL-1β, TNF-α, and IL-6 in the retina and vitreous humor through the suppression of the phosphorylation of STAT3 and NF-κB, and the binding of 67LR on the neurons and glia [65]. We also found similar ocular anti-inflammatory effects in the anterior chamber of the eye following LPS induction [39]. It ameliorated the expression of tumor necrosis factor-alpha (TNF-α), interleukin-6 (IL-6), and monocyte chemoattractant protein-1 (MCP-1) by CD43-positive leucocytes and CD68-positive macrophages and reduced the infiltration of leucocytes and macrophages into the iris and ciliary body. Our recent metabolomic analysis has shown that the ocular anti-inflammation caused by GTE was indirect through induced systemic phosphorylcholine lipids to suppress the inflammatory responses and alleviate the hepatic damage and mitochondrial stress [66]. Furthermore, GTE was able to attenuate uveitis on a murine model of experimental autoimmune uveoretinitis (EAU). It partially alleviated uveitis phenotypes and recovered visual function. GTE and EGCG are also able to down-regulate Th-17-associated pro-inflammatory genes, such as interleukin 1 beta (IL-1β), IL-6, IL-17A, and tumor necrosis factor-alpha (TNF-α) [67]. These findings provide evidence for the ocular anti-inflammatory effects of GTE and EGCG.

However, the bioavailability of EGCG is low, thereby limiting its capability for antioxidation and anti-inflammation treatments, especially for neural tissues and retina that are separated by various barriers. Nanotechnology may overcome such a limitation by a flavonoid-containing nanoparticle formulation [68]. EGCG-loaded liposomes enveloped with phosphatidylcholine or phosphatidylserine could improve the bioavailability. These liposomes attenuated LPS-induced pro-inflammatory cytokines and restored motor impairment in a Parkinsonian syndrome rat model, which was deemed to be possible through the inhibition of murine BV-2 microglial cells [69]. Meanwhile, EGCG has been per-acetylated as pro-EGCG to increase its tissue level and protect EGCG from oxidation before entering the cell [70]. Pro-EGCG is a potent anti-angiogenesis agent that acts against angiogenesis-dependent diseases, such as endometriosis [71]. In addition, the drug-delivery systems, such as encapsulation, can also be used to improve the stability and bioavailability of green tea catechins [72,73].

## 3. Pathophysiological Conditions in Glaucoma: Oxidative Stress and Inflammation

Glaucoma is a common and serious form of irreversible optic neuropathy, with abnormalities and dysfunction of the optic nerve estimated to affect over 100 million people by the year 2040 [74]. Glaucoma is characterized by the progressive loss of retinal ganglion cells (RGCs) and their axon, thinning of the retinal nerve fiber layer, cupping of the disc, and visual field defects [75]. The two major forms of glaucoma, primary open angle glaucoma (POAG) and primary angle closure glaucoma (PACG), are complex and multi-factorial in etiology involving genetic and environmental factors [76]. Risk factors for POAG include older age, elevated intraocular pressure (IOP), sub-Saharan African ethnic origin, positive family history, and high myopia. PACG is affected by older age, hyperopia, and east Asian ethnic origin [77]. Treatments based on topical eye drops, laser therapy, and surgical intervention to lower IOP is a clinically proven approach to prevent glaucoma progression [78]. RGC loss could arise happen in some patients who present with a good control of IOP [79]. RGCs are responsible for transmitting image-forming and non-image forming visual information from the retina to the brain. After optic nerve injury, activation of apoptosis, autolysis, pyroptosis, and ferroptosis, together with the early downregulation of autophagy and phagocytosis, are the major modes of cell death involved in RGC death [80]. Besides the modes of cell death, oxidative stress and inflammation are the major pathophysiological conditions that are implicated in the pathogenesis of glaucoma [81,82].

Dysregulation in the ocular blood flow is another major pathological factor in glaucoma. Unstable ocular blood flow causes chronic and repeated mild reperfusion, which induces the peroxynitrite and superoxide production in the astrocytes and the mitochondria of the RGCs [83]. Signs of chronic oxidative stress have been reported in the retinas from glaucomatous donors with increased levels of oxidative by-products compared to the control donors [81]. Increases in the superoxide dismutase (SOD) and glutathione peroxidase (GPX) activities were found in the aqueous humor of POAG and PACG patients compared to the cataract patients, while the levels of vitamin C and vitamin E were found to be significantly lower in the aqueous humor of POAG and PACG [84]. Moreover, lower levels of reduced and total glutathione were also found in POAG patients as compared to the control subjects adjusted for age and sex [85]. A lower redox index was found in the POAG patients than the age-matched controls [86]. Additionally, the immunostaining for hypoxia-inducible factor-1α (HIF1A), which is tightly regulated by the cellular oxygen concentration, was found to be increased in the retina and optic nerve head of glaucomatous donor eyes compared to the control eyes. The retinal location of the increased immunostaining for HIF1A was closely concordant with the location of the visual field defects recorded in some of the glaucomatous eyes [87].

Retinal microglia, the resident yolk sac-derived macrophage cells in the retina, act as the first and key active immune defense in the central nervous system, constantly scavenging for plaques, damaged or unnecessary neurons and synapses, and infectious agents [88]. Microglia are extremely sensitive to pathological changes to prevent pathological damage, including glaucoma-related stress. Their intricate interactions affect the diverse outcomes of the microglia–RGC relationship as either being neurosupportive or neurodestructive in nature [89]. Histological studies on human specimens indicated the proliferation of microglia in the optic nerve head from human donors with advanced glaucoma, including the lamina cribrosa, along with the upregulation of immunomodulating (transforming growth factor (TGF)-β2 and prostaglandin E2) and pro-inflammatory mediators (tumor necrosis factor (TNF)-α and inducible nitric oxide synthase) [90]. Moreover, increased levels of pro-inflammatory cytokines (TNF-α, interleukin (IL)-1β, IL-6, IL-8, and interferon (IFN)-γ) [91] as well as inflammasome components (NOD-like receptor pyrin (NLRP)-3 and caspase-1) [92] were reported in human glaucomatous eyes/retinas. Our previous animal experiments also demonstrated that acute IOP elevation upregulates inflammation protein marker (IL-1β, TLR-4, and TNF-α) expression in the rat retina [93].

**Figure 5:**
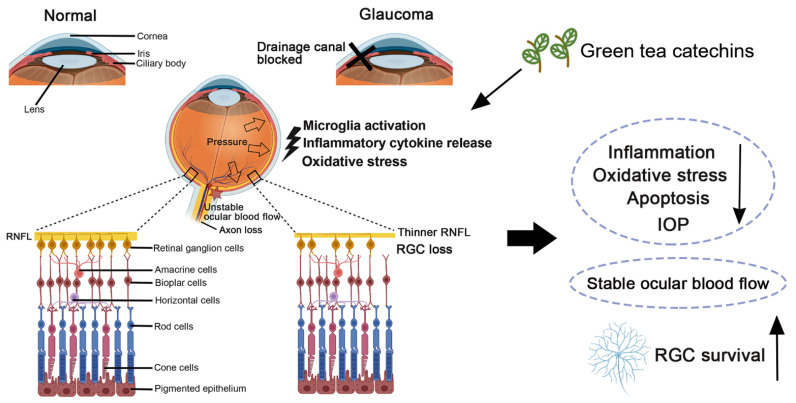
Pathophysiological conditions in glaucoma and the retinal ganglion cell protective effect of green tea catechins in glaucoma. RGC: retinal ganglion cell; and IOP: intraocular pressure.

There is thus abundant evidence supporting oxidative stress and inflammation in glaucomatous retina (Figure 5). Accordingly, oxidative stress and inflammation in the retina should be targeted for treatments in order to ameliorate RGC death in glaucoma.

## 4. Green Tea Catechins in Experimental Cellular Models of Glaucoma

Catechins attenuating oxidative stress and the inflammatory response could, in part, account for their neuroprotective capabilities [94]. To investigate the in vitro effect of green tea catechins, the primary culture of isolated RGCs [95], human stem cell-derived RGCs [96,97], and the retinal explant culture [98] have been used as glaucoma-related platforms on RGCs. However, these platforms have not been adopted to study the in vitro effects of green tea catechins on RGCs. Instead, a transformed mouse cell line, RGC-5 [99], was adopted in cellular studies, although this cell line later was characterized as the mouse SV-40 T antigen-transformed photoreceptor cell line, 661 W [100]. Earlier studies have demonstrated that 50 μM EGCG significantly reduces the apoptosis and ROS production in RGC-5 cells caused by 400 μM hydrogen peroxide [101]. Consistently, EGCG (2.5–10 μg/mL) was found to be able to improve the survival of RGC-5 cells upon hydrogen peroxide and ultraviolet radiation insults [102]. Moreover, EGCG (IC_50_: 0.8 μM) was found to be able to attenuate the formation of thiobarbituric acid reactive substance formation, a measure of lipid peroxidation, as induced by 20 μM sodium nitroprusside in rat brain homogenates [103]. Similarly, an one-hour pretreatment of EGCG (50 μM) and epicatechin (EC; 50 μM) was able to attenuate rotenone-induced toxicity in RGC-5 cells and inhibit sodium nitroprusside-induced lipid peroxidation (EGCG IC_50_: 2.5 μM; EC IC_50_: 1.5 μM) [103]. EGCG at concentrations greater than 10 μg/mL has been proven to inhibit RGC-5 cell growth [102]. This is consistent with our previous study on green tea extract (Theaphenon E; ≥16.25 μg/mL) and EGCG (≥25 μM) attenuating cell proliferation and migration [104,105]. For immunomodulation, EGCG treatment can cause immunosuppressive alterations on human monocyte-derived dendritic cells by inducing cell apoptosis and suppressing cell surface molecules and antigen presentation [106]. Administration of EGCG can also increase IL-10 levels in cell culture supernatants [107].

## 5. Green Tea Catechins in Experimental Animal Models of Glaucoma

Green tea catechins are able to cross the blood-brain barrier [108]. Tea polyphenols can reduce oxidative stress and IOP and stabilize ocular blood flow [109]. Glaucoma and RGC-injury animal models have been applied to evaluate the treatment effects of green tea extract and EGCG on RGC survival after injury (Figure 5). In the study of N-methyl-D-aspartate (NMDA)-induced excitotoxicity, NMDA-treated rats received two-day prophylactic treatments of intraperitoneal EGCG injections (25 mg/kg) showed a higher cell density in the ganglion cell layer and thickness of Thy-1 immunoreactivity than those received intraperitoneal saline injections [110]. For the optic nerve axotomy model, intraperitoneal injections of 50 mg/kg EGCG at 30 min before axotomy, and at Day 2 and 4 after axotomy were able to attenuate RGC loss by 12% in rat retina along with reducing the upregulation of neuronal nitric oxide synthase and Bax protein expression, and further enhancing ERK 1/2 and Akt activation after axotomy [111]. Inhibition of the ERK and Akt pathways could attenuate the protection effects of EGCG on RGCs against axotomy injury. In the optic nerve crush model, optic nerve-injured rats treated with EGCG showed a significantly higher density of RGCs at Day 7, 14, and 28 post-optic nerve crush, respectively, compared to those treated with the vehicle. Furthermore, there was a significantly higher expression of the neurofilament triplet L protein observed in the optic nerve-injured rats treated with EGCG than those treated with the vehicle [112]. Similarly, our recent study demonstrated that rats with pre- or post-operative treatment of 275 mg/kg green tea extract (Theaphenon E) showed a higher RGC survival and axonal regeneration and improved pupillary light reflex post-optic nerve injury with the activation of Akt, Erk p42/44, and Stat3, as well as the downregulation of inflammation, apoptosis, and microglia activation genes, compared to the saline-treated rats [113]. Pre-treatment of 275 or 550 mg/kg green tea extract was also able to reduce the activation of microglia in rats with an optic nerve injury.

In the chronic IOP elevation model induced by microbead injection into the anterior chamber, the IOP-elevated mice fed with EGCG-supplemented drinking water showed a higher RGC density than those fed with normal drinking water at Day 15 and 27 post-injury [114]. Notably, in the acute IOP elevation model, intraperitoneal injections of 50 mg/kg EGCG at 30 min before ischemia injury (raising the IOP to 150 mm Hg for 60 min) was able to reduce RGC death by 10%. There were also improvements in the TUNEL-positive cells observed in the inner retina, and neuronal NOS and nicotinamide adenine dinucleotide phosphate diaphorase-positive cells in the rat retina at Day 3 post-injury with the downregulation of ischemia injury-induced glial fibrillary acidic protein and lipid peroxidation [115]. Similarly, the ischemia-injured (raising the IOP to 120 mm Hg for 45 min) rats that received the EGCG treatment were determined to be able to attenuate the reduction in the a-wave and b-wave amplitudes of the electroretinograms, decrease Thy1 and neurofilament-L expression, increase retinal caspase-3 and caspase-8 expression, and blunt the changes in the localization of the retinal Thy-1 and ChAT immunoreactivities [101]. EGCG present in the drinking water (0.5%, 200 mL/day for 3 days before ischemia injury and 5 days after ischemia injury) was also able to ameliorate the ischemia injury (120 mm Hg for 45 min)-induced thinning of Thy-1 and choline acetyltransferase immunoreactivities, reduce a-wave and b-wave amplitudes of the electroretinograms, and Thy1 and neurofilament-L expression in the rat retina [116]. Similarly, our previous study on an experimental acute IOP elevation rat model (110 mm Hg for 2 h) demonstrated the anti-oxidative and anti-inflammatory properties of the green tea extract on ischemia-injured RGCs such that the oral administration of green tea extract (Theaphenon E; 275 mg/kg, 4 times within the first 2 days after the injury) ameliorated ischemic injury-induced RGC apoptosis and promoted RGC survival by reducing caspase-3 and caspase-8 expression, p38 phosphorylation, and inflammation marker (Il1β, Tlr4, and Tnfa) expression, as well as enhancing Jak phosphorylation in the retina [116].

In addition to the studies conducted with rodents, a single intravenous injection dose of 15 mg/kg EGCG in saline was found to reduce the TUNEL-positive and high-mobility group box-1-positive cells in the retinal sections of the ischemia-injured (raising the IOP to 100 mm Hg for 60 min) New Zealand male rabbits with the nuclear translocation of Nrf2 and increase in HO-1 expression at 6 h after treatment [117]. Moreover, EGCG can act directly on RGC axons in *Xenopus* embryos to increase the number of growth cone filopodia that responded to extrinsic signals in a Sema3a-independent manner and led to a dramatic defect in the guided growth of RGC axons, whilst EGCG itself had no influence on RGC axon behavior in *Xenopus* embryos [118].

## 6. Clinical Applications of Green Tea Catechins for Glaucoma Treatments

Antioxidants, including green tea components, have been proposed as biotherapies for glaucoma prevention [119]. In young males with CrossFit training, green tea extract supplementation (two capsules once daily for six weeks; 250 mg green tea extract per capsule, containing 245 mg polyphenols (200 mg catechins, among which 137 mg EGCG, <4 mg caffeine, microcrystalline cellulose, and magnesium stearate) doubled the total antioxidant capacity in the venous blood test while also lowering the plasma concentration of lipid peroxidation products [120]. Regular consumption of moderate quantities of green tea could effectively modulate the antioxidant capacity in people subjected to oxidative stress, along with lowering the glucose, lipid, and uric acid levels [121]. A combined analysis from the Nurses’ Health Study and the Health Professionals Follow-up Study in the United States reported that higher intakes of flavonols and monomeric flavanols were nominally associated with a lower POAG risk, and consuming ~2 cups of tea per day was associated with an 18% lower POAG risk [122]. Consistently, the United States 2005–2006 National Health and Nutrition Examination Survey reported that the participants who consumed at least one cup of hot tea daily showed a 74% decreased odds of having glaucoma compared with those who did not consume hot tea [123]. However, the Korea National Health and Nutrition Examination Survey 2010 to 2011 reported no significant associations between the frequency of tea consumption during the past 12 months and the risk of POAG with adjusting for multiple covariates [124]. The Rotterdam Study in the Netherlands also found no significant associations between flavonoid intake and the risk of POAG [125].

There were 122 studies on EGCG and 890 studies on green tea in the registry of ClinicalTrials.gov at the time of writing this manuscript. There was one study on EGCG and five studies on green tea extract related to ocular health/disease (Table 3). A randomized, placebo-controlled, double-blind, cross-over design clinical trial on EGCG in Italy (clinicaltrials.gov identifier: NCT00476138) reported that POAG patients who received oral EGCG treatment (200 mg/day) for 3 months in addition to standard IOP-lowering therapy showed increases in the amplitude of pattern-evoked electroretinograms as compared to the baseline values or to the patients who received the placebo treatment [126]. The magnitude of the pattern-evoked electroretinogram amplitude increments after EGCG treatment was inversely related to the corresponding baseline amplitudes. However, standard automated perimetry did not show significant changes after EGCG treatment. In addition, a recent clinical study from Lithuania reported that young volunteers receiving 400 mg green tea extract or EGCG capsules showed significant reductions in IOP after 2 h in the green tea extract group and after 1 h in the EGCG group as compared to the baseline [127].

A 6 month randomized, placebo-controlled clinical trial study in Washington (clinicaltrials.gov identifier: NCT01646047) aimed to evaluate the effects of a multi-component dietary supplement (containing vitamin C, mixed tocopherols/tocotrienols, vitamin D, fish oil, lutein, zeaxanthin, pine bark extract, benfotiamine, green tea extract, and curcumin; two capsules per day) on the visual function and retinal structure of the patients with type 1 or type 2 diabetes without retinopathy, or with mild-to-moderate non-proliferative retinopathy [123]. The study reported that study subjects on active supplement had a significantly better visual function and displayed significant improvements in most serum lipids, high-sensitivity C-reactive protein, and diabetic peripheral neuropathy compared to those who received the placebo. However, no significant changes in retinal thickness, hemoglobin A1c, total cholesterol, and TNF-α were found. A follow-up double-blinded, randomized, placebo-controlled clinical trial study in Washington (clinicaltrials.gov identifier: NCT03866005) aimed to evaluate the effects of “Diabetes Visual Function Study” softgels (containing lutein, zeaxanthin, vitamins B1, B12, C, D, and E, lipoic acid, coenzyme Q10, resveratrol, EPA/DHA, Pycnogenol™, grape seed extract, green tea extract, and curcumin; two or four capsules per day) to standard anti-vascular endothelial growth factor therapy for the subjects with diabetic macular edema. Another follow-up open-label, single-arm clinical trial study in California and Oklahoma (clinicaltrials.gov identifier: NCT04117022), which was estimated to be completed at the end of 2022, aimed to evaluate the ability of the chromatic electroretinogram and the full-field flicker electroretinogram in detecting the changes in global retinal function in diabetic retinopathy patients with dietary supplement treatments (DVS formula, consisting of vitamins C, D3, and E, zinc oxide, eicosapentaenoic acid, docosahexaenoic acid, α-lipoic acid, coenzyme Q10, mixed tocotrienols/tocopherols, zeaxanthin, lutein, benfotiamine, N-acetyl cysteine, grape seed extract, resveratrol, turmeric root extract, green tea leaf, and Pycnogenol; 2 softgels per day for 6 months). In addition, a randomized double-blinded clinical trial study conducted in Massachusetts (clinicaltrials.gov identifier: NCT00718653) aimed to measure the macular pigments and plasma lutein concentrations in subjects with lutein (12 mg per day) plus green tea extract (200 mg per day) treatment. Although this study was stated as completed, no results from this study have been reported as of yet.

## 7. Summary, Challenges, and Future Prospects

The pathophysiological mechanisms for RGC degeneration in glaucoma are complex. Although oxidation and inflammation are the major insults to RGCs, multiple modes of cell death are involved in RGC loss after optic nerve injury [80]. Targeting oxidation and inflammation alone do not adequately rescue RGCs from glaucomatous degeneration. The combined treatment of neurotrophic factors with antioxidative and anti-inflammatory agents should generate pronounced therapeutic effects against RGC degeneration [128,129]. Our study on sodium iodate-induced retinal degeneration model demonstrated that green tea extract (Theaphenon E) showed better treatment effects than EGCG alone or custom-made catechin mixture with EGCG [130], indicating that other constituents in green tea extract also possessed neuroprotective effects on RGCs. Furthermore, EGCG has a poor bioavailability, which could therefore affect its therapeutic effects on disease treatment. To enhance the stability and bioavailability of EGCG, the prodrug of EGCG (pro-EGCG, EGCG octaacetate) could be useful [71,131]. Further research is needed to delineate the stability, bioavailability, and neuroprotective effects of each catechin and their constituents in green tea extract as well as their metabolites. Currently, there has only been one double-blinded randomized placebo-controlled clinical trial for EGCG on eye disease, and none for the sole green tea extract treatment. Whether green tea extract and catechins could be a therapeutic treatment prescribed for glaucoma patients still requires additional clinical trials to confirm its clinical applications in glaucoma and different eye diseases. It has been reported that an herbal product made of a dry aqueous extract of green tea containing 90% of EGCG (one tablet per day) was prescribed by an ophthalmologist to treat a glaucoma patient. However, green tea-related hepatotoxicity was suspected [132]. Therefore, the dosage and safety of green tea extract or EGCG treatment for glaucoma patients should be seriously studied. Nevertheless, as multiple pre-clinical studies have proven the efficacy of green tea extract and EGCG on ameliorating RGC degeneration, green tea catechins could be a potential co-adjuvant counteracting the oxidation and inflammation in RGCs for glaucoma management in addition to the IOP-lowering therapies.

## Figures and Tables

**Table 1:** Summary of the biological properties of green tea catechins.

Biological Properties	Mechanisms	References
(1) Pro-oxidation	Electron resonance within the phenolic moiety following abstraction of proton by ROS.	[15,16]
(2) Antioxidation/-reduction	High reduction potential of catechins compared to endogenous antioxidants; reducing and recycling the oxidized endogenous molecules.	[17]
(3) Anti-inflammation	Binding to 67LR to increase Tollip expression, which negatively regulates TLR signaling to suppress inflammatory mediators.	[18]
(4) Anti-tumor, antioxidation, and anti-inflammation	Binding to the active sites of p53 and changing the structural conformation to prevent ubiquitination by MDM2; retaining the biological level and activities of p53. Inhibiting the activation of the wild-type and some mutant EGF receptors in non-small cell lung cancer cell lines.	[19,21]
(5) Moderate redox, inflammation, and cell cycle	Binding to the EGF receptor to change the topology and block EGF to activate the receptor for subsequent inflammation activities.	[20]
(6) Generation of secondary messengers for vasodilation	Inhibiting anti-oxidative molecules, including the Trx/Trx receptor to increase ROS, which acts as a secondary messenger for various pathways Activating CaMKKβ to increase energy metabolism; elevating cytosolic calcium to increase nitric oxide production. Increasing cAMP to promote the phosphorylation of eNOS and vasodilator-stimulated phosphoprotein to cause vaso-relaxation. Activating AMPK to reduce endothelin-1 expression for vasodilation.	[22,24,25,26,27,28,29]

67 LR: 67 kDa laminin receptors; AMPK: adenosine 5′ monophosphate-activated protein kinase; cAMP: cyclic adenosine 5′ monophosphate; CaMKKβ: calcium/calmodulin-dependent protein kinase beta; EGF: epidermal growth factor; eNOS: endothelial nitric oxide synthetase; MDM2: murine double minute 2; ROS: reactive oxygen species; TLR: Toll-like receptor; Tollip: Toll-interacting protein; and Trx: thioredoxin.

**Table 2:** Pharmacokinetics of the catechins of different green tea extracts in different ocular compartments.

Maximum Concentration	GTE	GC	EGC	C	EC	EGCG	GCG	ECG
**C_max_ (nM)**								
Plasma	Sunphenon DCF-1	91.5 ± 57.4	754.9 ± 235.8	139.0 ± 57.0	1258.4 ± 294.0	310.4 ± 59.9	50.8 ± 10.4	159.1 ± 33.9
	Theaphenon^®^ E	530.8 ± 200.2	13718.0 ± 4948.0	2990.0 ± 1990.0	9143.0 ± 1912.0	6687.0 ± 4437.0	131.3 ± 91.7	443.8 ± 352.3
Aqueous humor	Sunphenon DCF-1	-	602.9 ± 116.7	127.4 ± 62.8	138.9 ± 58.5	13.2 ± 5.1	33.5 ± 20.4	47.8 ± 8.1
	Theaphenon^®^ E	246.9 ± 34.9	911.3 ± 250.5	98.3 ± 19.2	708.1 ± 127.8	284.4 ± 58.4	0.57 ± 0.98	26.5 ± 10.3
Vitreous humor	Sunphenon DCF-1	110.6 ± 22.1	15.9 ± 7.0	96.5 ± 23.3	20.5 ± 10.6	15.4 ± 2.7	20.9 ± 9.9	14.0 ± 5.1
	Theaphenon^®^ E	4492.0 ± 443.5	404.1 ± 102.5	321.7 ± 69.5	436.8 ± 102.5	2224.4 ± 805.4	33.9 ± 31.0	369.6 ± 74.0
**C_max_ (ρmol/g)**								
Choroid–sclera	Sunphenon DCF-1	11461.8 ± 5168.7	1506.3 ± 941.1	477.6 ± 346.9	283.5 ± 66.5	184.4 ± 39.0	220.5 ± 69.7	10.7 ± 4.3
	Theaphenon^®^ E	188.28 ± 111.3	542.2 ± 335.1	294.7 ± 32.8	1818.0 ± 563.0	1183.0 ± 611.0	59.0 ± 54.8	518.0 ± 292.0
Retina	Sunphenon DCF-1	22729.4 ± 4229.4	8020.8 ± 1658.5	492.7 ± 235.2	608.0 ± 112.0	259.1 ± 67.2	3.2 ± 1.9	-
	Theaphenon^®^ E	61.0 ± 43.5	118.2 ± 55.6	35.7 ± 15.0	174.5 ± 45.8	784.4 ± 195.9	59.0 ± 54.8	64.0 ± 16.0
Lens	Sunphenon DCF-1	1558.1 ± 318.4	1172.3 ± 207.8	300.0 ± 151.5	72.3 ± 19.1	149.1 ± 26.5	18.0 ± 6.6	90.3 ± 45.8
	Theaphenon^®^ E	1.9 ± 3.0	10.9 ± 8.9	4.1 ± 4.8	4.6 ± 6.9	43.9 ± 25.8	0.4 ± 0.6	1.0 ± 3.0
Cornea	Sunphenon DCF-1	-	359.4 ± 66.8	58.5 ± 15.4	30.6 ± 5.7	25.2 ± 15.5	10.7 ± 3.9	91.1 ± 18.7
	Theaphenon^®^ E	10.8 ± 16.7	59.5 ± 26.7	61.7 ± 17.5	536.4 ± 61.1	634.6 ± 122.9	18.8 ± 24.2	101.8 ± 43.1
**Elimination**	**GTE**	**GC**	**EGC**	**C**	**EC**	**EGCG**	**GCG**	**ECG**
**λz (h^−1^)**								
Plasma	Sunphenon DCF-1	0.107 ± 0.010	0.213 ± 0.015	0.104 ± 0.038	0.371 ± 0.000	0.236 ± 0.007	0.171 ± 0.013	0.211 ± 0.010
	Theaphenon^®^ E	0.270 ± 0.030	0.390 ± 0.040	0.370 ± 0.080	0.400 ± 0.050	0.230 ± 0.020	1.250 ± 0.380	0.210 ± 0.040
Aqueous humor	Sunphenon DCF-1	-	0.045 ± 0.001	0.209 ± 0.012	0.093 ± 0.062	0.304 ± 0.012	0.111 ± 0.033	0.124 ± 0.043
	Theaphenon^®^ E	0.110 ± 0.020	0.240 ± 0.020	0.130 ± 0.030	0.210 ± 0.040	0.090 ± 0.020	-	0.130 ± 0.120
Vitreous humor	Sunphenon DCF-1	0.166 ± 0.010	0.041 ± 0.001	0.106 ± 0.030	0.067 ± 0.004	0.058 ± 0.012	0.042 ± 0.006	0.224 ± 0.035
	Theaphenon^®^ E	0.020 ± 0.010	0.110 ± 0.090	0.110 ± 0.060	0.100 ± 0.030	0.080 ± 0.020	-	-
Choroid–sclera	Sunphenon DCF-1	0.057 ± 0.001	0.461 ± 0.015	0.220 ± 0.014	0.488 ± 0.007	0.267 ± 0.019	0.929 ± 0.049	-
	Theaphenon^®^ E	-	0.250 ± 0.090	0.220 ± 0.090	0.370 ± 0.060	0.080 ± 0.040	-	0.150 ± 0.070
Retina	Sunphenon DCF-1	0.188 ± 0.045	0.203 ± 0.050	0.245 ± 0.010	2.432 ± 0.154	0.413 ± 0.040	-	-
	Theaphenon^®^ E	-	0.040 ± 0.030	0.040 ± 0.010	0.060 ± 0.020	0.040 ± 0.020	-	0.090 ± 0.030
Lens	Sunphenon DCF-1	0.302 ± 0.049	0.084 ± 0.020	0.234 ± 0.032	0.049 ± 0.004	0.269 ± 0.011	3.160 ± 0.130	-
	Theaphenon^®^ E	-	-	-	-	0.130 ± 0.060	-	-
Cornea	Sunphenon DCF-1	-	0.170 ± 0.031	0.116 ± 0.007	0.043 ± 0.012	0.125 ± 0.001	0.372 ± 0.006	0.477 ± 0.021
	Theaphenon^®^ E	-	-	0.220 ± 0.100	0.220 ± 0.100	0.090 ± 0.020	-	0.100 ± 0.090

GTE: green tea extract; GC: (−)-gallocatechin; EGC: (−)-epigallocatechin; C: (+)-catechin; EC: (−)-epicatechin; EGCG: (−)-epigallocatechin-3-gallate; GCG: gallocatechin-3-gallate; and ECG: (−)-epicatechin-3-gallate.

**Table 3:** Registered clinical trials of green tea extract or EGCG application for eye diseases.

Identifier.	Country	Status	Phase	Enrollment	Targeted Eye Diseases or Conditions	Intervention	Dosage	Duration
NCT00476138	Italy	Unknown	Phase I/II	40	Primary open angle glaucoma Ocular hypertension	Oral EGCG treatment	200 mg/day	3 months
NCT00718653	United States	Completed	Not Applicable	40	Eye health	Lutein plus green tea extract	Lutein (12 mg/day) Green tea extract (200 mg/day)	Unknown
NCT01646047	United States	Completed [118]	Not Applicable	70	Diabetes Mellitus—Type 1 Diabetes Mellitus—Type 2 Non-proliferative diabetic retinopathy	Multi-component nutritional supplement capsules (vitamin C, mixed tocopherols/tocotrienols, vitamin D, fish oil, lutein, zeaxanthin, pine bark extract, benfotiamine, curcumin, and green tea extract)	2 capsules/day	6 months
NCT02984813	United States	Terminated	Phase I	21	Open-angle glaucoma Diabetic retinopathy	Nutritional supplements capsules (alpha lipoic acid, citicoline, Co-enzyme Q10, Ginkgo biloba extract, grape seed extract, N-acetyl-cysteine, curcumin, and green tea extract)	2 capsules/day	3 months
NCT03866005	United States	Unknown	Not Applicable	150	Center-involved diabetic macular edema	Multi-component nutritional supplement capsules (macular carotenoids lutein, zeaxanthin, vitamins B1, B12, C, D, E, lipoic acid, coenzyme Q10, resveratrol, patented extract of French maritime pine bark grape seed, curcumin, and green tea extract)	2 or 4 capsules/day	Study duration
NCT04117022	United States	Recruiting	Not Applicable	45	Diabetes Diabetic Retinopathy	Multi-component nutritional supplement capsules (vitamins C, D3 and E (d-α tocopherol), zinc oxide, eicosapentaenoic acid, docosahexaenoic acid, α-lipoic acid, coenzyme Q10, mixed tocotrienols/tocopherols, zeaxanthin, lutein, benfotiamine, N-acetyl cysteine, grape seed extract, resveratrol, turmeric root extract, Pycnogeno, and green tea leaf)	2 capsules/day	6 months

Information obtained from http://clinicaltrials.gov/ (accessed on 27 June 2022). EGCG: (−)-epigallocatechin-3-gallate.
